# Supplementary material for: N-Methyl-d-glucamine–Calix[4]resorcinarene Conjugates: Self-Assembly and Biological Properties
Source: Molecules. 2019 May 20;24(10):1939. doi: 10.3390/molecules24101939 (PMC6572135; doi:10.3390/molecules24101939)
Supplement: Supplementary file 1 [file molecules-24-01939-s001.pdf]

molecules

Article

N-methyl-D-glucamine–Calix[4]resorcinarene Conjugates: Self-Assembly and Biological Properties

Ruslan R. Kashapov [1,2,*], Yuliya S. Razuvayeva [1,2], Albina Y. Ziganshina [1], Rezeda K. Mukhitova [1], Anastasiia S. Sapunova [1], Alexandra D. Voloshina [1], Victor V. Syakaev [1], Shamil K. Latypov [1], Irek R. Nizameev [1,2], Marsil K. Kadirov [1,2] and Lucia Y. Zakharova [1,2]

[1] Arbuzov Institute of Organic and Physical Chemistry, FRC Kazan Scientific Center of RAS, 8 Arbuzov str., Kazan 420088, Russia; kashapov@iopc.ru

[2] Kazan National Research Technological University, 68 K. Marks str., Kazan 420015, Russia

* Correspondence: kashapov@iopc.ru; Tel.: +7843-273-22-93; Fax: +7843-273-22-53 (F.L.)

Supplementary materials

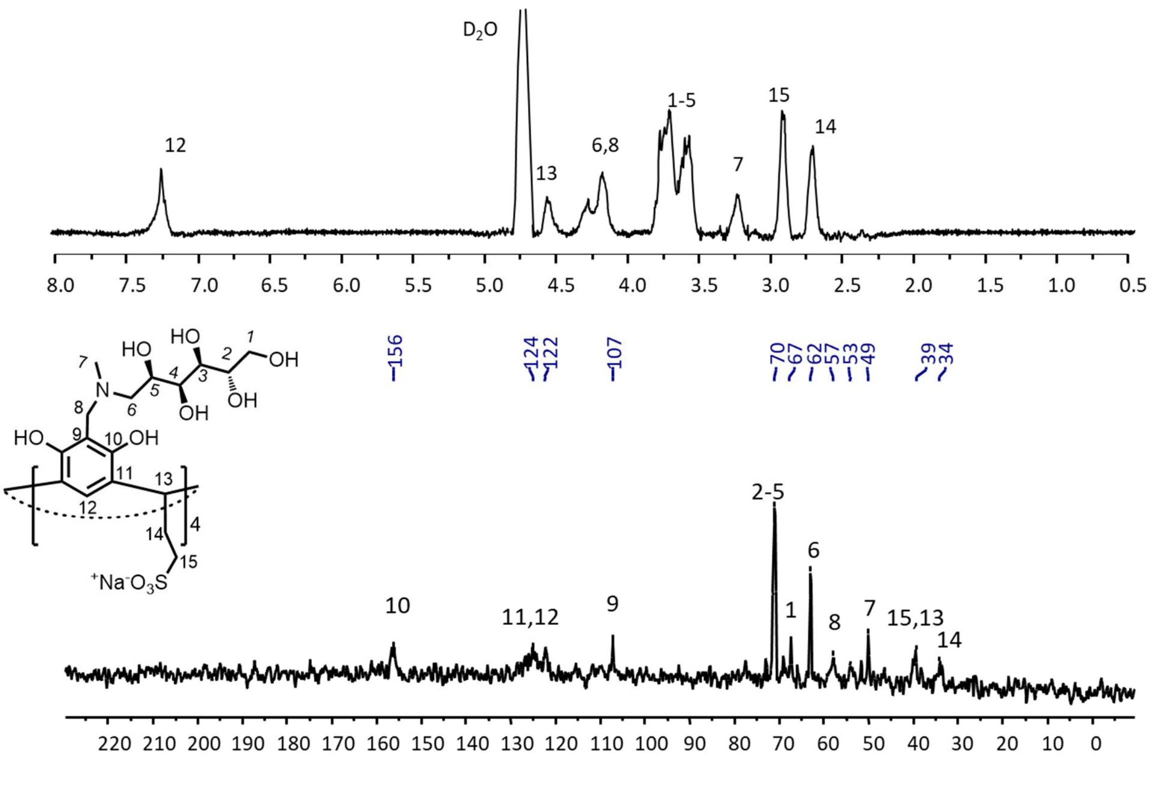

Figure S1. ^{1}H and ^{13}C NMR spectra of GCR-1.

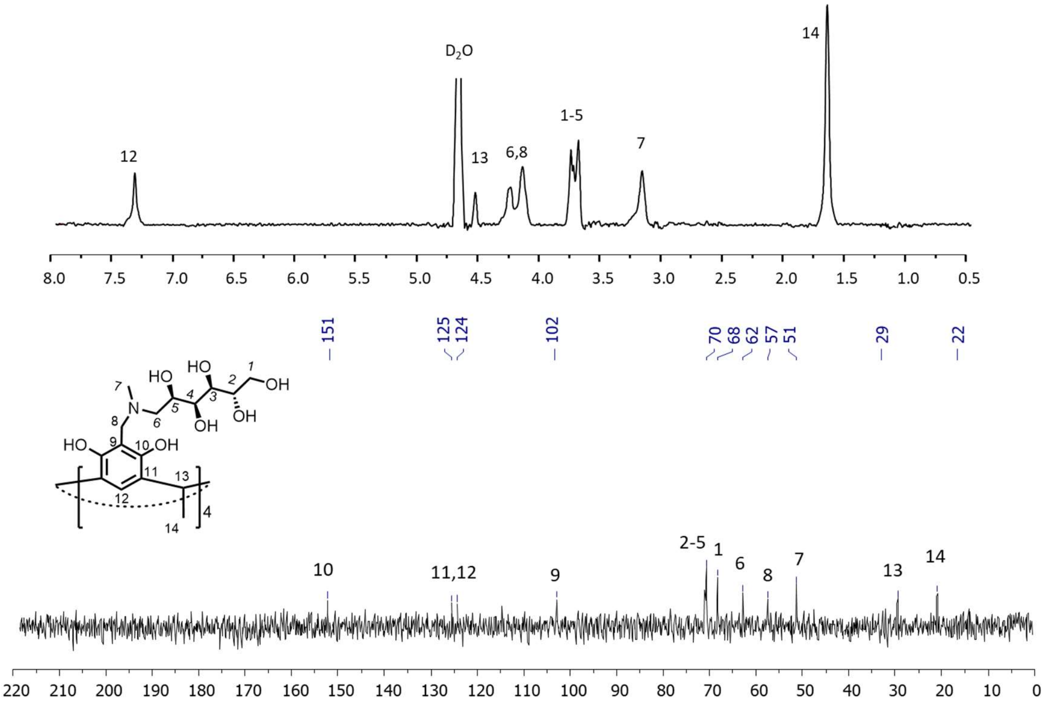

Figure S2. ^{1}H and ^{13}C NMR spectra of GCR-2.

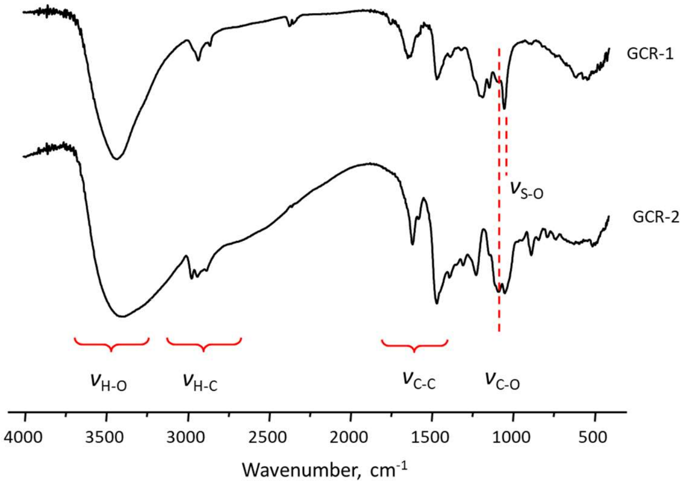

Figure S3. IR spectra of GCR-1 and GCR-2.

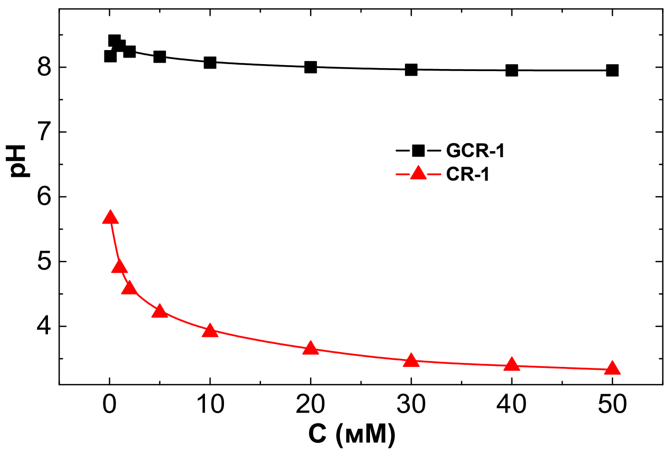

Figure S4. Concentration dependence of pH of water solutions of CR-1 and GCR-1, 25°C.

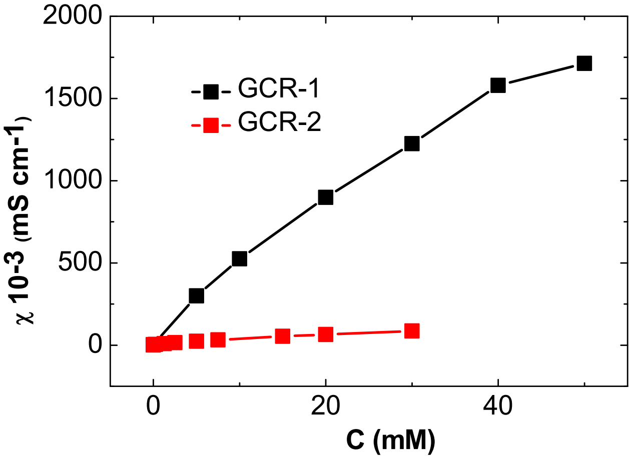

Figure S5. Concentration dependence of specific conductivity (χ) of GCR-1 and GCR-2 in 50% DMSO – 50% water, 25°C.

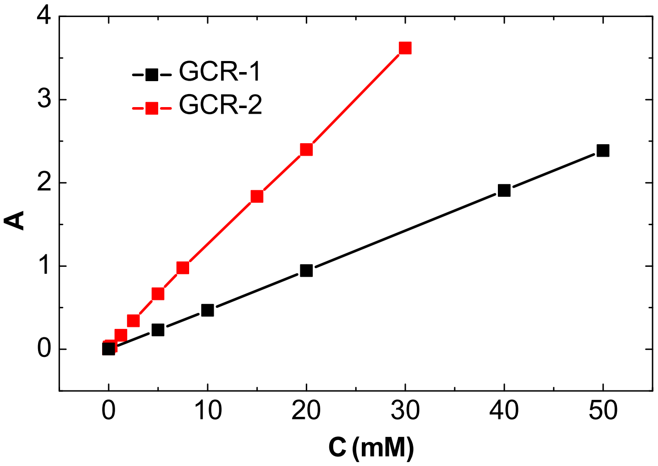

Figure S6. Concentration dependence of absorption at 500 nm for GCR-1 and GCR-2 in 50% DMSO – 50% water, 25°C.
